# First-Principles Evaluation of Proton Hopping in Tetrahedral Oxide Motifs

**DOI:** 10.1021/acs.chemmater.5c02422

**Published:** 2026-02-23

**Authors:** Shenli Zhang, Andrew J. E. Rowberg, ShinYoung Kang, Joel B. Varley

**Affiliations:** † Quantum Simulations Group, Materials Science Division, 4578 Lawrence Livermore National Laboratory, Livermore, California 94550, United States; ‡ Laboratory for Energy Applications for the Future, Lawrence Livermore National Laboratory, Livermore, California 94550, United States

## Abstract

Proton-conducting oxides (PCOs) are important materials used as ionic conductors for energy conversion technologies. Existing research efforts on PCO optimization and discovery generally focus on complex perovskite-based oxides that require doping and alloying to engineer oxygen deficiency and high proton conductivity. However, the variety of chemical compositions and coordination environments in oxides poses challenges for efficient materials design. In this computational study, we construct a database of simplified motifs to elucidate the relationship between fundamental materials chemistry and proton kinetics. Specifically, we focus on the zincblende crystal structure as a proxy for tetrahedral metal–oxide (*M*–O) coordination environments. We systematically quantified the effects of cation type, oxidation states, and *M*–O bond lengths on the proton hopping barrier, and found that strong *M*–O bonds and metal cations with large and variable oxidation states (e.g., Mo^6+^, V^5+^) lead to smaller proton hopping barriers. By mapping the candidate cations and their preferred bond geometries onto materials databases such as the Inorganic Crystal Structure Database (ICSD) and Materials Project, we identified real materials containing the corresponding metal–oxide units. In general, we observed good agreement between the calculated proton hopping barriers obtained in real crystal structures and those predicted by our motif database. We also discuss the limitations of our model and possible future extensions to improve its predictive capabilities. Overall, our model provides a first step for the rational design and quick screening of energy-efficient PCOs.

## Graphical abstract

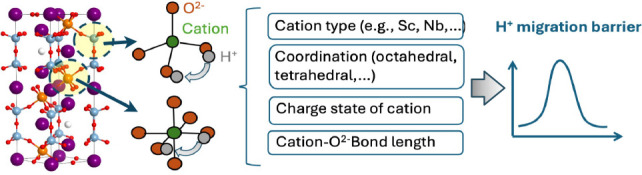

## Introduction

Proton-conducting oxides (PCOs) are important materials for various energy conversion and information technologies,^1^ including neuromorphic devices,^2,3^ solid oxide fuel cells (SOFCs), and solid oxide electrolyzer cells (SOECs).^4,5^ Comparing to oxide-ion conductors, systems based on PCO electrolytes offer lower operation temperature (typically between 300 and 700 °C, compared with 700–1000 °C for oxide-ion conductors)^6^ and thus lower energy consumption. This is because the activation energy for protons to diffuse is generally lower than that for oxide ions.^7^

The perovskites barium cerate (BaCeO_3_, BCO) and barium zirconate (BaZrO_3_, BZO), along with their derivatives, are the most widely studied PCOs due to their high proton conductivities (up to 0.01 S/cm at 600 °C by adjusting the Ce/Zr ratio in BZO–BCO alloys and incorporating dopants^8–12^ ). However, the incorporation of protons into BZO and BCO requires the initial presence of oxygen vacancies, which act as seeds for protonation when exposed to water. Vacancy formation is typically realized through acceptor doping; however, this requirement increases complexity in fabrication and device performance optimization while also increasing device variability. Acceptor doping also has several notable consequences for the device performance. For one, depending on the dopant and operating conditions,^5,13^ it can increase unwanted electrical leakage,^14,15^ which limits the Faradaic efficiency and overall performance of devices. Another concern is the electrostatic binding interaction between negatively charged acceptor dopants and positively charged protons, which is often large enough to impede proton diffusion significantly, particularly at the high doping concentrations often used experimentally.^16–18^ One solution to these challenges is to use materials that can incorporate hydrogen without doping while also maintaining favorable properties for proton transport.

There are numerous oxide systems that can circumvent this doping requirement due to their intrinsic oxygen deficiency. Many of these materials additionally exhibit high ionic conductivity comparable to the conventional perovskites.^7,19,20^ These include hexagonal perovskites, which contain mixed stacking sequences of hexagonal and cubic close-packing of [AO_3_] layers, as well as brownmillerite structures, which are ordered vacancy compounds based on the traditional ABO_3_ perovskite structure with 0.5 O atoms missing per formula unit (ABO_2.5_). Some examples of highly conductive hexagonal perovskites are Ba_7_Nb_4_MoO_20_, with proton conductivity $\sigma_{H^{+}}=4\times 10^{-3\;}S/\mathrm{cm}$ at 500 °C;^21^ Ba_5_Er_2_Al_2_ZrO_13_, with $\sigma_{H^{+}}=3\times 10^{-3\;}S/\mathrm{cm}$ at 500 °C;^22^ and Ba_2_ScAlO_5_, with about $\sigma_{H^{+}}∼10^{-3\;}S/\mathrm{cm}$ above 300 °C^23^). Among brownmillerite materials, HSrCoO_2.5_ displays unusually high proton conductivity, $\sigma_{H^{+}}=0.028-0.33\;S/\mathrm{cm}$ between 40 °C and 140 °C^24^). Interestingly, many of those structures are composed of tetrahedral moieties, which undoubtedly play a key role in dictating proton and oxide ion kinetics. Previous studies conclude that the high flexibility and rotational mobility intrinsic to these tetrahedral units allow for delocalization of the proton (and, similarly, oxide ions) and, correspondingly, low-energy diffusion pathways.^19,20,25^ Furthermore, certain elements are repeatedly represented within tetrahedral units in these PCOs, including Al, Mo, and Nb, strongly suggesting that the local geometry and chemistry influence the proton conductivity.

As proton transport obeys a Grotthuss-like transport mechanism^1^ (hopping between neighboring oxygen sites), and the proton conductivity follows an Arrhenius-like dependence, modifying the proton hopping barriers via the design of local motif chemistry should tune the proton conductivity. The challenge, however, is that within complicated crystal structures with varied coordination environments and multiple cation elements it is difficult to isolate and identify the impact of each descriptor.

Here, to address this problem, we build a database of cation-oxygen motifs to clarify the relationship between local chemistry and bond length and the proton hopping barrier, focusing specifically on *M*O_4_ tetrahedral coordination environments. As illustrated in Figure 1, we chose zincblende as a representative crystal structure containing repeated, corner-sharing *M*O_4_ units. The closely related wurtzite crystal structure was also used for model validation. This simplified, uniform, and controllable chemical environment allows us to investigate the impact of different geometrical and chemical descriptors on the proton hopping barrier. Focusing on 16 representative cations that commonly appear within *M*O_4_ units in real materials,^26^ we evaluated the hopping barrier of protons within a tetrahedral unit with density functional theory (DFT) calculations. We clarified the impact of the metal element, its oxidation state, and *M*–O bond length on the proton hopping barrier to establish structure–chemistry–property relationships underpinning proton transport in these model systems. Our results identified Mo^6+^, W^6+^, V^5+^, and Nb^5+^ as the most promising cations for facile proton hopping, and we investigated the sensitivity of associated barriers to their local bond lengths. To validate the effectiveness of our motif model, we further map several metal ions (Mo^6+^ and V^5+^) and their preferred bond geometries onto the Materials Project database^27^ to identify real materials containing analogous *M*O_4_ units. We found generally good agreement between the proton hopping barriers calculated in real crystal structures and those predicted by our motif database, while noticing that our motif model tends to underestimate the hopping barrier for certain transition metals in complex structures, where proton transport is governed by the interplay between different coordination environments. Overall, our model serves as a first step to unravel the design principles of novel oxides and to screen for their selection for efficient proton-conducting and energy conversion applications.

**Figure 1:**
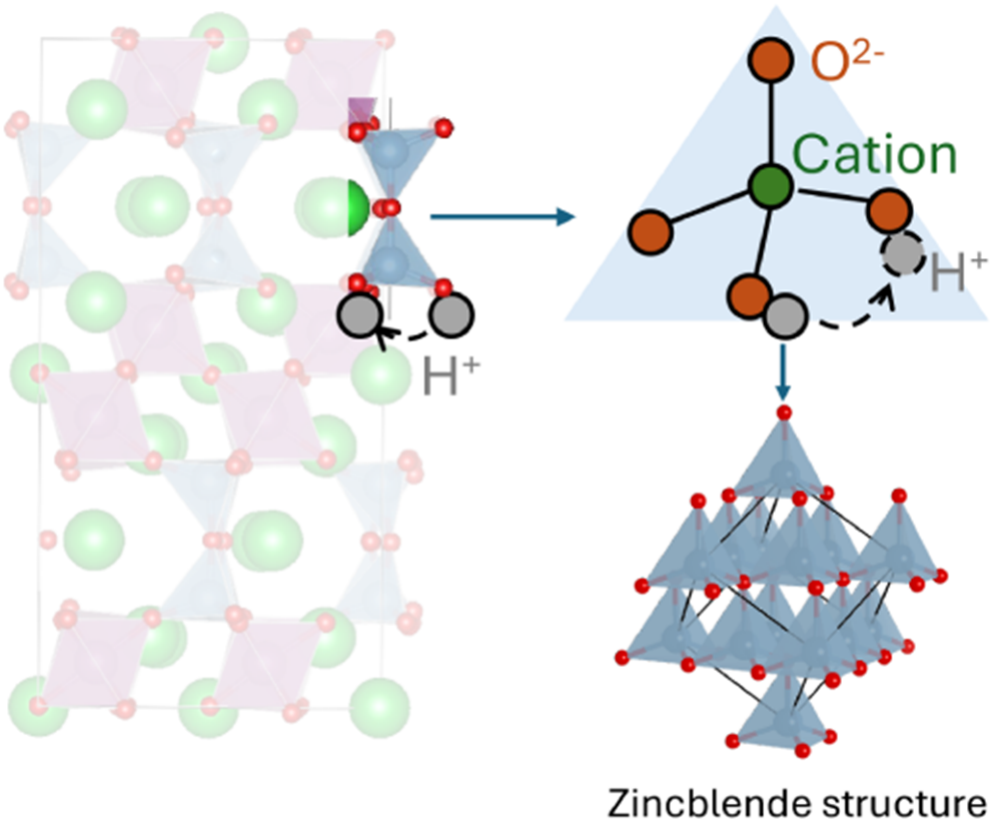
Schematic representation detailing our approach to isolate tetrahedral *M*O_4_ motifs from complex crystal structures, then calculating proton hopping barriers as a function of chemistry (cation element *M* and its oxidation state) and bond length.

## Methods

DFT calculations^28,29^ were performed using the Vienna *Ab initio* Simulation Package (VASP)^30–33^ v5.4.4, with the Perdew–Burke–Ernzerhof (PBE) exchange-correlation functional^34^ selected for computational efficiency. We chose the projector augmented wave method (PAW) pseudopotentials^35,36^ in VASP (potpaw.54), using “_pv” or “_sv” (if no “_pv” was available) valence configurations for transition metal ions, while for other elements the standard pseudopotential files were used. An energy cutoff of 500 eV was applied for all of the calculations. To control the oxidation state of the metal elements, and to ensure that hydrogen incorporates as a proton $\left(H_{i}^{+}\right)$ , instead of as the physically unrealistic neutral species,^37^ we set the total number of electrons in the system to a target value matching our chemical intuition. For example, in the case of the hypothetical AlO zincblende structure, we take out 1 electron per formula unit (f.u.), allowing Al to maintain its preferred 3+ oxidation state, and we remove one additional electron when hydrogen is included. We note that the removed electron (or the equivalent hole) is localized on one of the metal ions closest to hydrogen for In, Sn, W, and Mo cases, while it is delocalized for other cases. The localization behavior seems to be correlated with the low electron affinity of the elements. We verified that the background charge introduced in our structure should have minimal impact on the results, as a supercell size test on Al^3+^ (64-atom cell vs 216-atom cell) shows less than 0.01 eV change in the proton hopping barrier. All calculations are non-spin-polarized. While this choice can have a significant impact on the underlying electronic structure for particular oxidation states where varying orbital occupation and magnetic ordering have a strong influence on the ground state configuration, previous work has shown that the magnetic configuration (antiferromagnetic or ferromagnetic) has a very small influence on calculated migration barriers, justifying this simplification.^38^ We performed tests for several element and oxidation state choices to evaluate this sensitivity, finding that spin-polarization did not influence the calculated migration barriers and ground states for systems of interest like Mo^6+^, W^6+^, V^5+^, and Nb^5^. We did find a larger impact for certain cases, like Co, where tests for non-spin-polarized vs (001) and (111) antiferromagnetic ordering on the cation lattice showed significant impacts on the total energies, but were found to have a relatively small impact on the calculated barriers. For example, we found an average decrease of 0.08 eV for 2 evaluated lattice constants for Co^3+^ for the antiferromagnetic orderings relative to non-spin-polarized calculations. Additionally, the oxidation state of transition metals can also have another major impact on the proton hopping barrier: with certain oxidation states, the motif structure becomes metallic, and the proton will directly bond to transition metals, mimicking the behavior of a hydride anion $\left(H_{i}^{-}\right)$ . We excluded such cases from our database and limited our discussions to insulating phases, where the proton only hops between oxygen sites.

For each zincblende motif structure (including different oxidation states), an equilibrium *M*–O bond length was obtained by Birch–Murnaghan equation-of-state fitting to a series of self-consistent calculations at different lattice parameters. The same equilibrium bond length was assumed for the wurtzite structure. For zincblende structures, a unit cell of 4 *M*O f.u. was used, with an 8 × 8 × 8 Γ-centered **k**-point mesh. The obtained equilibrium bond lengths can be found in Table S1 in the Supporting Information (SI).

To obtain the proton hopping barrier, we performed climbing-image nudged elastic band (NEB) calculations^39,40^ with VASP, using a 2 × 2 × 2 zincblende structure (32 f.u.) (or 4 × 4 × 2 wurtzite structure, 64 f.u.) and a 4 × 4 × 4 **k**-point mesh. As the artificial motif structures may be dynamically unstable and undergo nonphysical global distortions during proton hopping for certain metal elements, we fixed the positions of all metal ions, except for the ones connected to oxide ions involved in the proton hopping process during NEB calculations. In Figure S1, we compare the proton hopping barrier obtained with metal cation positions fixed and allowed to relax freely for Al^3+^-, Ga^3+^-, and In^3+^-containing structures, finding that fixing cation positions may lead to a small, near-constant upward shift of the hopping barrier curve (within 0.1 eV for Al and Ga, and about 0.2 eV for In). While this energy difference may be significant for low proton hopping barriers, the qualitative relationship between the proton hopping barrier and the local geometry and chemical environment remains intact, meaning that this method should be suitable for systematic comparisons across different elements. We note that in this work we only focus on intrahopping barriers of *M*O_4_ units, although we acknowledge that rotational modes of these units may also contribute to proton migration.^41,42^

## Results and Discussion

### Impact of Bond Length and Chemistry on Proton Hopping Barrier

In Figure 2, we show the change of proton migration barrier for different cation elements and oxidation states as a function of *M*–O bond length using the zincblende crystal structure. The plots are grouped according to the corresponding group or versatility of typical oxidation states of the transition metals under analysis. We note that only selected oxidation states are presented for transition metals on the plot (e.g., only Ni^3+^ instead of Ni^2+^ is shown), for two reasons: one, not all oxidation states can be stabilized in tetrahedral coordination environments based on a representative chemical and structural analysis by Waroquiers et al. on a database of observed oxides;^26^ and two, some oxidation states result in hydrogen bonding to metals rather than oxygen (e.g., forming $H_{i}^{-}$ as described in the Methods section). All hopping pathways occur between two neighboring O sites, as shown schematically in Figure 1 (see Figure S2 for another example of the hopping pathway and its energy profile extracted from an NEB calculation). The range of the tested *M*–O bond lengths was determined by the stability of the motif structure. Specifically, no significant distortion of the tetrahedral units (see example in Figure S3 or *M*–O bond breakage should occur during proton hopping; our observation of tetrahedral distortions therefore determines the minimum possible bond length, while the scission of *M*–O bonds determines the largest such length.

**Figure 2:**
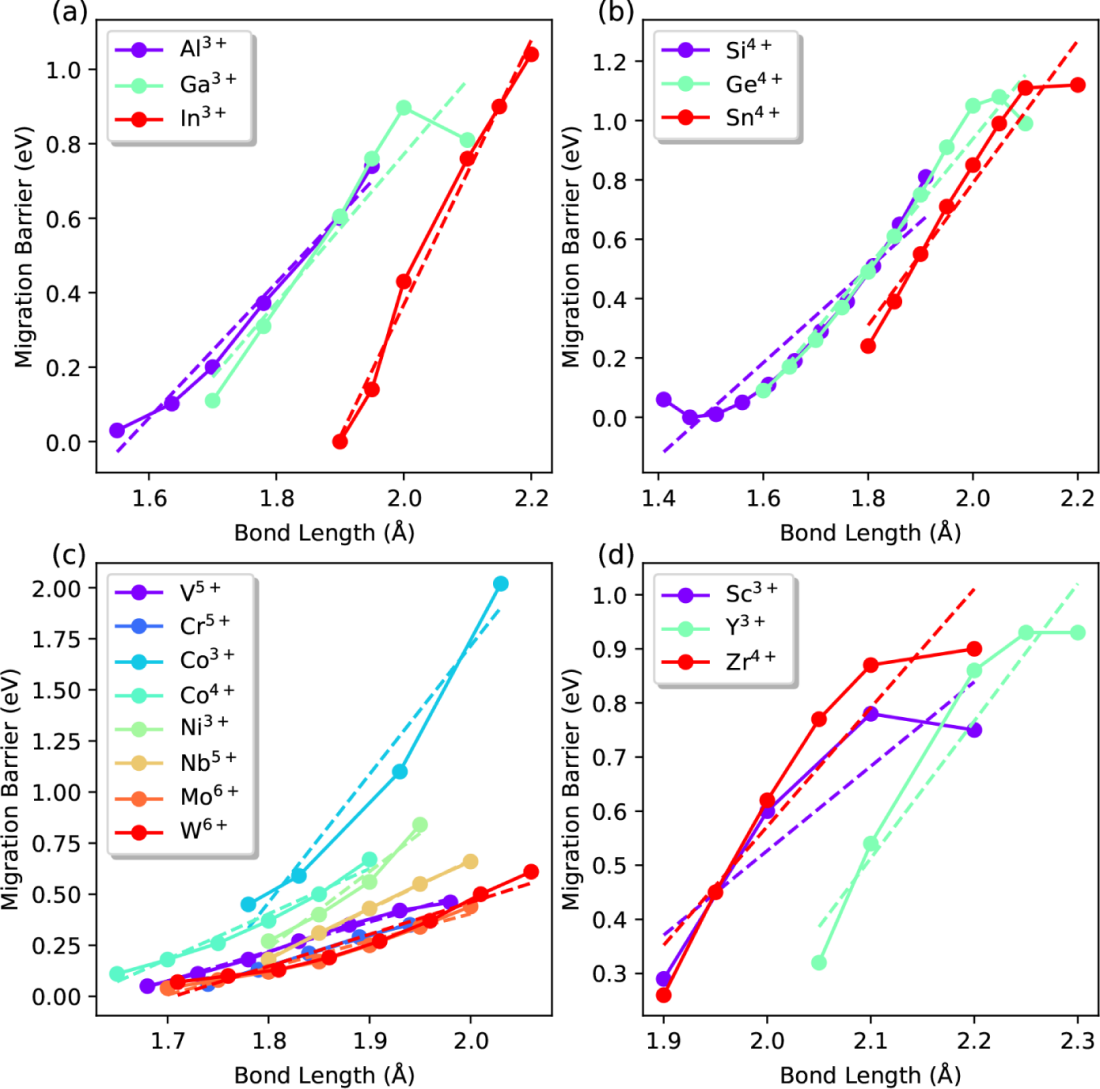
Computed proton migration barrier as a function of *M*–O bond length using the zincblende motif structure. The dashed line represents linear fitting for the data points. (a) Group III elements. (b) Group IV elements. (c) Transition metals that demonstrating multiple oxidation states according to ref. 26. (d) Transition metals with one dominant oxidation state according to ref. 26.

For most elements, the equilibrium *M*–O bond length (Table S1) is only slightly longer than the shortest *M*–O bond length shown in Figure 2, which helps to anchor the *M*–O bond length to a reasonable starting value. We found that the proton migration barrier generally increases with the *M*–O bond length, although it decreases slightly near the longest bond lengths for some elements (specifically, Ga^3+^, Ge^4+^, Sn^4+^, Sc^3+^, Y^3+^, and Zr^4+^). The overall trends obtained for the barrier vs bond length were found to be well described by a linear (the dashed lines in Figure 2) fit rather than a higher-order polynomial fit. The coefficients for these fits, including the slope, *y*-axis intercept, *x*-axis intercept, and the square of the correlation coefficient *R* ^2^, are included in Table S2. Specifically, the slope reflects the sensitivity of the hopping barrier to bond length change, while the *x*-axis intercept shows the bond length that will lead to barrier of 0 eV. The fitted parameters here serve as important indicators to select candidate materials and will be discussed more in detail below. The decrease of the hopping barrier at longer bond length can be understood by the concurrent change of *M*–O bond strength and hopping distance with increasing *M*–O bond length: the increase of *M*–O bond length decreases the *M*–O binding strength, while favoring the O–H bond, and thus increases the migration barrier; at the same time, the decrease of *M*–O bond strength also increases the bending of O–*M*–O at the transition state, which facilitates proton hopping (see Figure S4 for visualization). At a certain *M*–O bond length, the latter effect dominates, and the migration barrier starts to decrease. In fact, the competition between the two factors is also shown in the migration barrier change as a function of hopping distance (calculated as the distance between the initial and final proton positions, see Figure S5). The hopping distance starts to decrease beyond a certain bond length, and it is possible to have a similar hopping distance at shorter and longer bond lengths. Their migration barriers, however, are not equivalent. The migration barrier at the same hopping distance from a larger bond length is higher, due to the decreased *M*–O bond strength and a favored O–H bond, as mentioned above.

Ideally, the optimal cation element for fast proton transport should provide a low proton migration barrier over a wide range of *M*–O bond lengths, which is reflected by a small slope with increasing bond length in the figure (the dashed lines in Figure 2). Several transition metal ions, including W^6+^, Mo^6+^, V^5+^ and Nb^5+^, display such behavior. It is important to note that the slope of the migration barrier as a function of bond length depends on the chemical environment, i.e., the type of cation element and oxidation state. First, we find that all transition metal cations with easily variable oxidation states, shown in Figure 2c, exhibit smaller slopes compared to other cation groups. This tendency could originate from their flexible ability to adjust oxidation states during bond formation and breakage as well as the bond distortions experienced during proton hopping. The impact of oxidation state is reflected by comparing the cases of Co^3+^ with Co^4+^, and by noting, in general, that more positive oxidation states lead to smaller changes in the migration barrier with increasing bond length. This phenomenon can be explained by the stronger *M*–O binding with a higher oxidation state (also reflected by shorter equilibrium *M*–O bond length; see Table S1), which decreases the O–H binding strength and thus the proton migration barrier. In addition, the stronger electrostatic repulsion between protons and highly electropositive cations presumably plays a significant role in reducing barriers. Similarly, with increasing ionic radius or decreasing electron affinity in the same cation group (e.g., from Al^3+^ to In^3+^ in Figure 2a, and from Si^4+^ to Sn^4+^ in Figure 2b), the slope tends to increase, as the *M*–O binding strength decreases (again supported by longer equilibrium *M*–O bond length in Table S1), while the opposite trend is expected for O–H binding strength.

The inverse relationship between *M*–O binding strength and migration barrier is further supported by changing O^2–^ to N^3–^, where N^3–^ is expected to have weaker binding strength with *M*.^43^ H migration barriers in *M*–N compounds are correspondingly found to be higher than in *M*–O compounds (Figure S10), although we note this is likely not universal across all structures and chemistries. In summary, Figure 2 reveals that strong *M*–O bonds and the availability of flexible oxidation states are two key factors that can facilitate the easy proton migration. We also considered the chemical hardness^44^ as a possible electronic structure descriptor for the proton hopping barrier, which represents the chemical stability of an element and can be calculated as half of the band gap energy. Our results in Figure S6 show 1) for a fixed cation element, the proton hopping barrier decreases with increasing chemical hardness, since this quantity is larger at shorter *M*–O bond length; and 2) when comparing among different cation elements, each element has a specific range of chemical hardness, while the corresponding proton hopping barrier range and the rate of change can be similar. We thus conclude that chemical hardness may not be the ideal descriptor here.

We also emphasize that the *M*–O bond length appears to be the most straightforward geometric descriptor for the proton migration barrier change among several other descriptors we examined, as summarized in Figure S7. We investigated the correlation of the proton migration barriers to other variables such as the O–H bond length, proton hopping distance (the distance between the initial and final proton positions), *M*–O–*M* bond angle, and mass-weighted displacement of the hydrogenated center relative to the pristine structure. We found that despite some general trend connecting the migration energy to the proton hopping distance and O–H bond length descriptors (Figures S5 and S8), their mathematical relationships are more complicated than that corresponding to the *M*–O bond length descriptor. For the *M*–O–*M* bond angle (Figure S9) and the mass-weighted displacement of the hydrogenated center, there is no clear correlation with the proton migration barrier. Thus, the linear relationship between the *M*–O bond length and the proton migration barrier is an important finding of our study.

### Mapping Motif Calculations to Real Crystal Structures

Having identified the properties of tetrahedral metal–oxygen units that can lower the proton hopping barrier, we now search the Materials Project Database^27^ to find example materials that incorporate optimal tetrahedral units. Specifically, for each element and oxidation state, we can obtain a distribution of *M*–O bond lengths from the crystal structure database used in ref. 26, as shown in Figure 3 (the bond length distributions for other elements are shown in Figure S16). This database contains about 8000 unique, experimentally observed compounds that were evaluated for each cation site in the primitive unit cells to analyze local tetrahedral coordination environments, tabulating every cation–O bond length for these sites. The same compound can however repeatedly appear for two different *M*–O local environments, e.g., for multi-cation oxides that exhibit different tetrahedrally coordinated cations like Al^3+^ and Si^4+^ in Mg_2_Al_4_Si_5_O_18_ (Materials Project ID mp-6174). Coupling our calculated motif barriers with the mean *M*–O bond lengths for a certain element, we can determine the average proton hopping barrier expected for tetrahedral units of different metals. We find that Mo^6+^ tends to have the lowest average proton hopping barrier (Figure 3). Furthermore, by identifying the bond lengths leading to the lowest proton migration barriers (e.g., materials exhibiting the smallest *M*–O tetrahedral bond lengths), we can search within the crystal structure database for materials that contain tetrahedral units with a targeted cation element, *M*–O bond length, and oxidation state.

**Figure 3:**
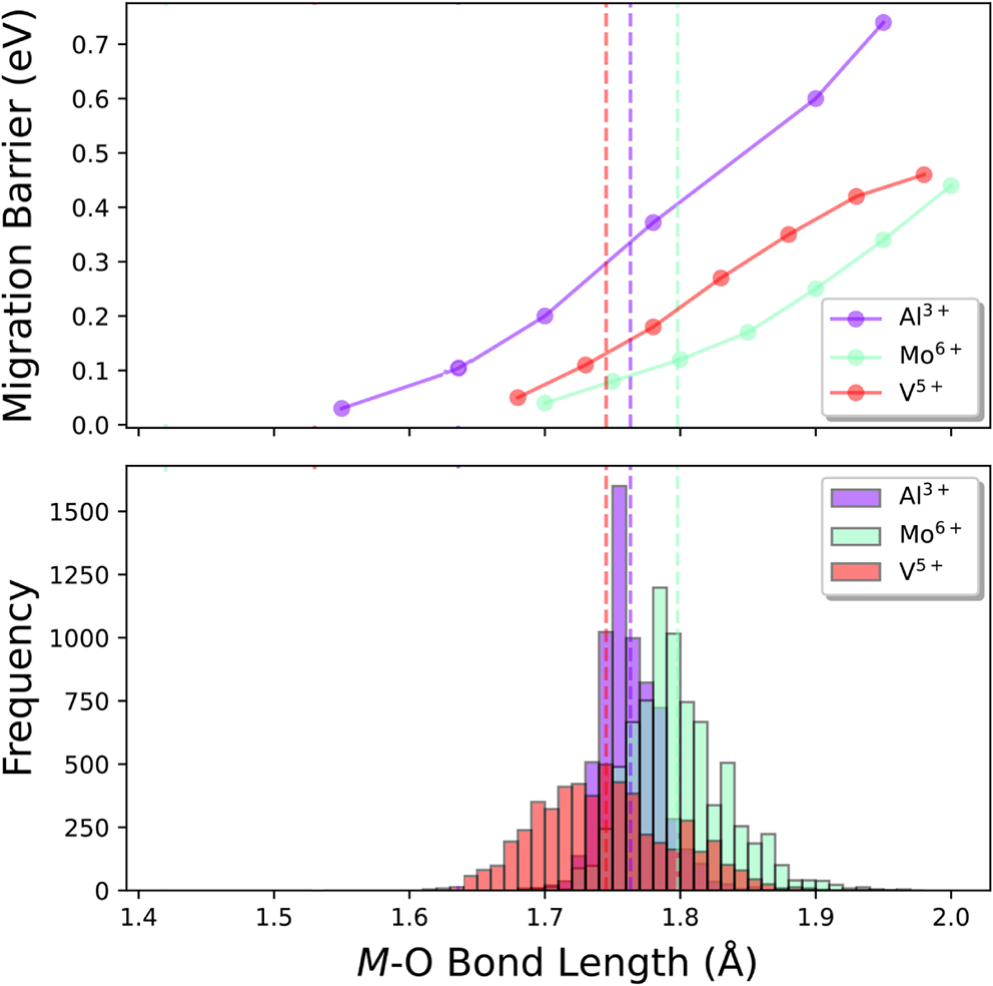
Distribution of *M*–O bond lengths in tetrahedral units in real materials obtained from crystal structure databases,^26,27^ using Al^3+^, Mo^6+^, and V^5+^ as examples. The corresponding proton migration barrier at the same *M*–O bond length is included for reference. Dashed vertical lines represent the mean of the distribution. The total counts in the histograms of the *M*–O bond lengths reflect 4 bond length entries for every tetrahedrally coordinated cation site in the primitive unit cells of structures containing a given cation and oxidation state.

Our analysis identified several crystal structures satisfying these criteria, such as Ba_3_V_2_O_8_ and Sr_3_V_2_O_8_, which contain tetrahedrally bonded V^5+^ with a V–O bond length around 1.72 ± 0.01 Å, as well as a family of alkali (*A*) catena-vanadate *A*VO_4_ structures such as the orthorhombic CsVO_4_ (space group 57, Materials Project ID mp-504651) and analogs with Rb and K that exhibit average tetrahedral V–O bond lengths of 1.73 ± 0.07 Å. The analysis also identified Mo^6+^-containing compounds like orthorhombic Zr­(MoO_4_)_2_ (space group 31, mp-636731) and trigonal Hf­(MoO_4_)_2_ (space group 167, mp-6870901), as shown in Figure 4, which feature tetrahedral Mo^6+^ with average Mo–O bond lengths of 1.76 ± 0.04 Å. Ba_3_V_2_O_8_ represents a family of palmierite oxides with VO_4_ units that have previously been associated with favorable proton conductivity,^42^ while the *A*VO_4_, Zr­(MoO_4_)_2_, and Hf­(MoO_4_)_2_ compounds have not been investigated for ionic conduction to the best of our knowledge. We also considered other promising proton-conducting oxides with common tetrahedral cations, including β-Ba_2_ScAlO_5_ and Ba_5_Er_2_ZrAl_2_O_13_ (Figure 4, containing Al^3+^ tetrahedral units), and Ba_7_Nb_4_MoO_20_ (Figure 4d, containing Nb^5+^ and Mo^6+^ tetrahedral units), to compare proton migration barriers in real crystal materials with those from our zincblende motifs.

**Figure 4:**
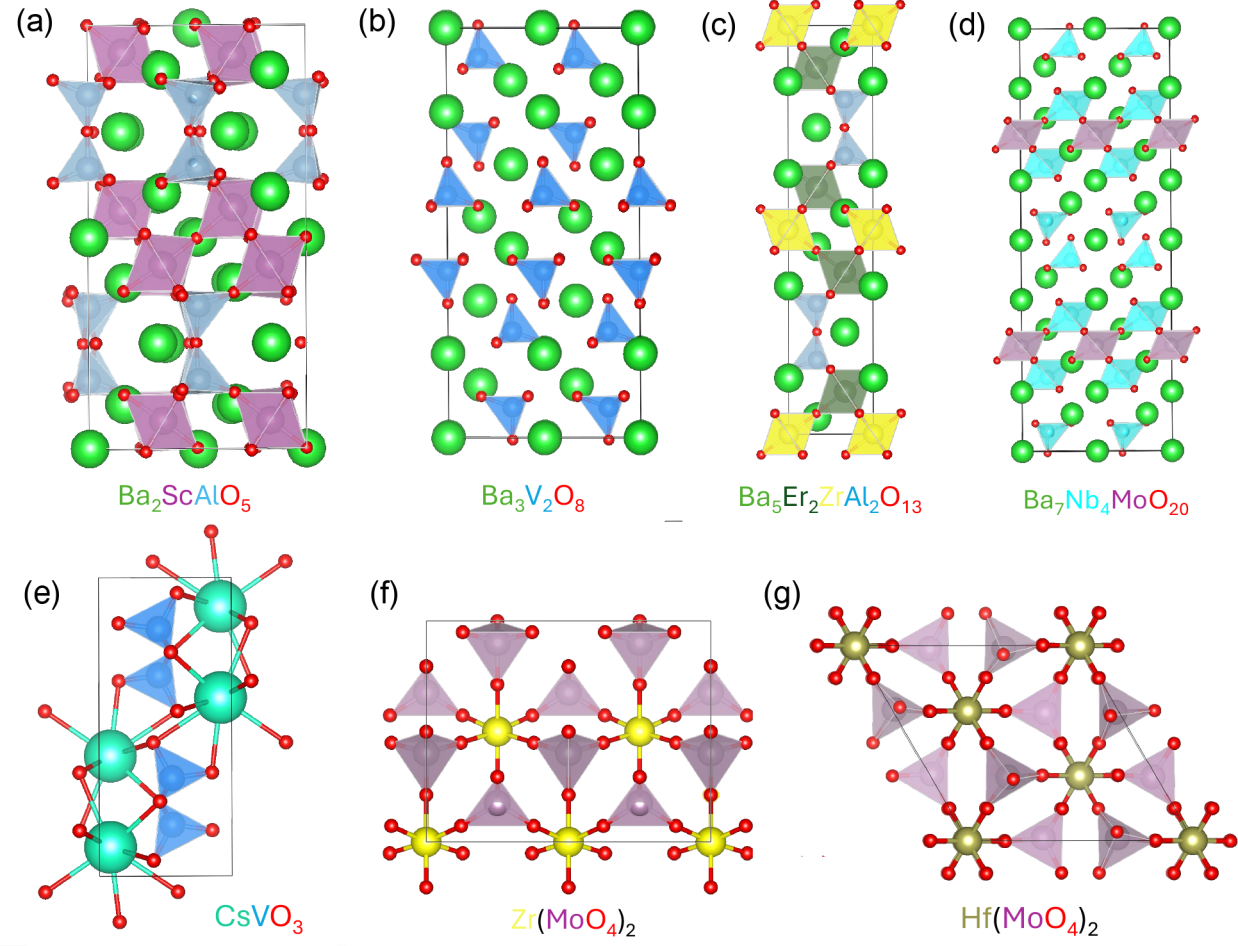
Selected crystal structures to compare the proton hopping barrier in the corresponding tetrahedral units with the value in our motif model structures. (a) β-Ba_2_ScAlO_5_, (b) Ba_3_V_2_O_8_, (c) Ba_5_Er_2_ZrAl_2_O_13_, (d) Ba_7_Nb_4_MoO_20_, where Nb and Mo occupy the same site, (e) CsVO_3_, (f) Zr­(MoO_4_)_2_, and (g) Hf­(MoO_4_)_2_. All structures were obtained from the Materials Project database, except for β-Ba_2_ScAlO_5_ and Ba_7_Nb_4_MoO_20_, where we adopted the structures obtained from neutron diffraction data and X-ray diffraction data in refs. 23 and 45 respectively, using the Bilbao Crystallographic Server.^46,47^ The atomic coordinates for the two structures are provided at the end of SI. Structural representations are visualized using the VESTA3 software.^48^

We calculated the intrahopping barrier (hopping around one tetrahedral unit, instead of across different units, which we refer to as interhopping) for protons in Zr­(MoO_4_)_2_, Ba_3_V_2_O_8_, β-Ba_2_ScAlO_5_, Hf­(MoO_4_)_2_, and CsVO_3_ with the NEB method, with the lowest proton hopping barriers summarized in Table 1. We also include the literature references, where available, for both calculated proton migration barriers and measured activation energies for proton conduction. The details for each calculated proton hopping pathway can be found in Figures S11–S14 and Tables S3 and S4. By taking into account the variation of energy barriers within the reference data themselves (reported as mean absolute deviation (MAD) and mean values in Table 1), we compared the migration barrier in the motif structure and the mean barrier obtained in the compound structures, and found the difference is small (0.07 and 0.27 eV, respectively) for Al^3+^ and Nb^5+^, while it is bigger (0.32 and 0.76 eV, respectively) for the Mo^6+^ and V^5+^ cases,. The small difference for the Al^3+^ and Nb^5+^ cases indicates that insights from highly local coordination environments can be matched with reasonable accuracy to much more complex structures. The increased difference for Mo^6+^, however, is partially due to the inclusion of the global activation energy for proton transport in the reference data, which leads to the highest deviation, as it likely includes the effects of other proton hopping processes. We also note another reason for the discrepancy comes from the difficulty of one-to-one comparison between our motif structure and compound structures, because we found multiple hopping pathways and associated barriers can manifest for one tetrahedral unit in the studied materials due to varied *M*–O bond lengths and different neighboring environments that result from having inherently more complex structures than our model systems (e.g., multiple Wyckoff positions for symmetrically distinct cation and O sites within the lattice). For example, in the case of CsVO_3_, some oxygen ions in the VO_4_ tetrahedral unit are shared between V sites, while the others are shared between Cs sites, whereas in Ba_3_V_2_O_8_ there are two unique O Wyckoff positions (O1 and O2) that exhibit different bond lengths and associated proton binding affinities within 0.1 eV for the same VO_4_ unit. For Zr­(MoO_4_)_2_ and Hf­(MoO_4_)_2_, only interhopping pathways (hopping between different tetrahedral units) are possible. Nevertheless, our motif model consistently underestimates the proton hopping barrier compared to that of real materials. The applicability and potential limitations of our motif model for realistic proton barrier estimation are discussed in detail below.

**Table 1:** Proton Migration Barrier Comparison: Comparison of Migration Barriers of Tetrahedrally Coordinated Elements Obtained in Motif Zincblende Structures to Those Obtained in Real Crystal Structures (Only the Lowest Migration Barrier Is Shown Here) with Similar Bond Lengths^a^

Cation	Structure	Bond length (Å)	Migration barrier (eV)	Activation energy (eV)	MAD, mean, diff to mean
Al^3+^	motif	1.78	0.37	–	0.10, 0.44, −0.07
	β-Ba_2_ScAlO_5_	1.78	0.3	0.64 ^23^	
	Ba_5_Er_2_ZrAl_2_O_13_	1.74–1.78	0.41 ^49^	0.40 ^22^	
Mo^6+^	motif	1.8	0.12	–	0.09, 0.44, −0.32
	Zr(MoO_4_)_2_	1.70–1.78	0.38	–	
	Ba_7_Nb_4_MoO_20_ ^b^	1.79	–	0.57,^21^ 0.40 ^50^	
	Hf(MoO_4_)_2_	1.77–1.95	0.36	–	
V^5+^	motif	1.73	0.11	–	0.28, 0.87, −0.76
	Ba_3_V_2_O_8_	1.71–1.72	1.1, 0.3 ^42^	0.99–1.07 ^42^	
	CsVO_3_	1.96	1.04	–	
Nb^5+^	motif	1.85	0.32	–	0.02, 0.59, −0.27
	Ba_7_Nb_4_MoO_20_ ^b^	1.82	0.61	0.57,^21^ 0.40 ^50^	

a Activation energy barriers, typically based on experimental measurements of ion (i.e.proton) conductivity, are also included for reference, although we note that these values reflect global proton transport in these materials, which is not limited to specific proton hopping processes (e.g., hopping around a tetrahedral site). Mean absolute deviation (MAD) and mean values of all computed and literature values in compound structures (including both migration and activation energy barriers) are provided. This is without including the value obtained from the motif structure. The difference between the migration barrier obtained in the motif structure and the mean value of the barrier in the compound structure is also provided (Diff to mean).

b Site-specific migration barriers for Mo–O and Nb–O tetrahedra have not previously been computed, although one study reports a migration barrier of 0.61 eV for proton migration near a tetrahedral site containing a cation vacancy, computed using the PBEsol functional;^25^ ref. 23: activation energy is for below 300 °C based on experimental measurement of $\sigma_{H^{+}}$ ; ref. 22: based on total ionic conductivity measured by experiments under wet condition from 300 to 450 °C; ref. 49: CI-NEB calculation with the PBE functional; ref. 21: experimental activation energy for bulk below 300 °C; ref. 42: bond-valence site energy (BVSE) calculations for the migration barrier; experimental activation energy based on total conductivity under dry and wet conditions.

### Discussion on the Applicability of Our Motif Model

First, we note that in a uniform chemical environment the proton hopping barrier seems to be determined mainly by the local structural features. To be specific, we obtained similar migration barriers in the wurtzite crystal structure as for the zincblende structure, using Al^3+^ as an example (Figure S1). This finding indicates that the results for some promising cations identified in our simplified motif model may be generalized to a broader range of crystal structures incorporating tetrahedral units.

Second, although the PBE functional we used here is expected to underestimate the proton migration barrier due to its delocalization error, the trend of the proton migration barrier change as a function of bond length and chemical environment should remain valid even for higher levels of theory. We found a similar migration barrier slope for Sc^3+^ obtained using the SCAN functional,^51^ although the migration barrier values are somewhat larger than those computed with PBE (see Figure S15).

Finally, there are some limitations in matching proton hopping barriers from our simplified motif model to those in more complex systems. For example, in Zr­(MoO_4_)_2_, we found that protons tend to avoid intrahopping pathways, even when the initial and final oxygen sites are attached to the same Mo atom. We found that one of the Mo sites (“Mo_2_” in Figure S11) tends to form a fifth bond with an oxygen atom attached to a neighboring Mo site when protons are present, providing a bridging site for the proton to hop back and forth (see Figure S11). The Mo atom to which the proton moves (“Mo_1_”) cannot form this MoO_5_ unit, meaning that protons will be trapped there, and only repeated interhopping will occur. Certain paths may result in hydrogen binding to metal cations as hydride species and lead to unrepresentative hopping barriers (all of the hopping barriers are reported in Table S3).

In a similar fashion, for Ba_7_Nb_4_MoO_20_, proton hopping around Mo–O tetrahedra is highly improbable. As can be seen in Figure 4(e), Ba_7_Nb_4_MoO_20_ is composed of octahedral and tetrahedral layers, with the former exclusively comprised of Nb–O octahedra, and the latter consisting of disconnected Nb–O and Mo–O tetrahedra (i.e., no O atom is shared between adjacent tetrahedra). We attempted to stabilize protons in coordination with each symmetrically inequivalent oxygen site in the lattice, but for O atoms bonded to Mo, the protons spontaneously relax to O sites coordinated to Nb instead. Furthermore, even when bonded to Nb–O tetrahedra, protons within the tetrahedral layer of Ba_7_Nb_4_MoO_20_ are approximately 1.1 eV higher in energy than protons contained within the Nb–O octahedral layer. Consequently, barriers for proton diffusion within the tetrahedral layer are large and highly anisotropic: we compute intrahopping barriers for Nb–O tetrahedra of 1.29 and 1.75 eV for protons moving away from and toward the center of the tetrahedral layer, respectively. Subsequent migration of a proton from the tetrahedral layer into the octahedral layer proceeds with a much smaller 0.41 eV barrier compared with 1.04 eV from an octahedral O into the tetrahedral layer. We note, however, that interstitial oxygen defects will be readily accommodated within the tetrahedral layer.^52^ These defects bridge Mo–O and Nb–O tetrahedra—in a similar fashion to the case described above for Zr­(MoO_4_)_2_—and protons can bind with them favorably^25^ in an interstitial hydroxide configuration. These hydroxide species are relatively low in energy and may form during hydration. Thus, the tetrahedral layer may be key to introducing protons into Ba_7_Nb_4_MoO_20_, but once present, they will likely move rapidly into the octahedral layer and conduct around Nb–O octahedra, much like in a typical perovskite oxide. This illustrates the limitations of a simplified tetrahedral site model, where even in layered materials with tetrahedrally coordinated *M*O_4_ linker species, migration pathways can be more complicated due to details of the local and global structural and chemical environment. The preference of the proton incorporation at the NbO_4_ sites over MoO_4_ sites, as well as variations in proton affinity even within a given *M*O_4_ unit with varied local environments within the lattice, both suggest the importance of evaluating the absolute and relative proton formation energies at different interstitial sites as important features for improving the generality of our model. Thus, more detailed consideration of proton formation energies is necessary in future studies for both informing predictions on overall proton hopping pathways in more complex chemical and structural environments as well as providing insight into hydration activity.

All of the above observations indicate that, in a complex material environment, Mo^6+^ may not form O–H bonds easily, likely due to its highly positive oxidation state, which will repel positively charged protons, and its strong Mo–O bond strength. Thus, the extremely low proton hopping barriers we obtained for certain cations with high oxidation states may not be realistic in more complex materials, where H–O bonds will form more favorably in proximity to less electropositive elements. This point can explain why, in Table 1, the same motif units (e.g., AlO_4_ and MoO_4_) can have highly dissimilar proton hopping barriers in complex crystal structures due to the variation in the *M*–O bond length and bond strengths influenced by the neighboring environment. The relatively large discrepancy in migration barriers obtained in the VO_4_ motif and Ba_3_V_2_O_8_ crystal structure may also be due to the impact from neighboring environment. Thus, a distribution of proton hopping barriers, rather than a single value based on our motif model, should be considered when designing oxides for fast proton conductivity. The general underestimation of the proton hopping barrier in our motif structure compared to the values in real materials is no doubt a consequence of neglecting the heterogeneous neighboring environment. We also note that our current selection of the candidate materials in Figure 4 is based on kinetic factors only, where we neglected the impact of thermodynamics for proton incorporation into the materials and the role of oxygen deficiency on hydration activity. This should be an important factor to consider for future comprehensive screening of promising candidates and whether intrinsic oxygen deficiency is an important factor to consider for proton incorporation in those candidate materials. We therefore intend in future work to expand our analysis to more complex migration pathways as well as adding thermodynamic considerations, thereby capturing the effects of mixed coordination more accurately.

## Conclusions

In summary, we have used the simplified zincblende crystal structure to construct repeating tetrahedral *M*–O motifs to investigate the impact of local geometry and chemistry on proton hopping barriers. Overall we find that the *M*–O bond length exhibits the most reliable correlation with the hopping barriers in our model structures, identifying that strong metal–oxygen bonds (e.g., short *M*–O bond lengths) and the presence of metal cations with high and variable oxidation states lead to smaller barriers. By mapping several promising metal ion candidates such as Mo^6+^ and V^5+^ and their preferred bond geometries onto the Materials Project database, we identified and assessed existing materials containing the corresponding metal-oxide units, some of which had not been previously considered as proton conductors. We generally found a good agreement within ∼0.2 eV between the proton hopping barriers calculated in real crystal structures and those predicted by our motif model, showing that this screening approach may be useful for designing or optimizing favorable proton transport in complex materials with tetrahedrally coordinated species.

That said, this simplified motif model does not capture all factors influencing proton mobility in more complex crystal structures, including the importance of the neighboring environment. Unrealistic *M*–O binding strengths and hopping barriers may be predicted when relying solely on the model, particularly in cases in which tetrahedra are disconnected and where multiple cations are tetrahedrally bonded to oxygen. Adding complexity by analyzing proton migration in more diverse cation networks and including other motif models such as those for *M*–O octahedral units should be prioritized to extend our approach to increasingly complex systems. We expect a similar increasing trend of proton hopping barriers with increasing bond distance in octahedral units, which we observed for model rock salt structures (Figure S17), while the proton hopping barrier in the octahedral units is likely to be smaller than in the tetrahedral units at the same *M*–O bond length due to smaller effective hopping distance between two oxygen sites for cations with higher coordination numbers. Nevertheless, the work outlined here provides a recipe for understanding the importance of highly local structural features to overall proton conduction in both existing and novel oxides.

## Supplementary Material



## Notes

Equilibrium metal–oxygen bond length for calculated elements; Linear fitting parameters for proton hopping barrier change with metal–oxygen bond length; Impact of fixed configuration on proton hopping barrier; Impact of other descriptors, including hopping distance, chemical hardness, O–H distance, and M–O–M bond angles, on the proton hopping barrier; Comparison of proton hopping barrier with different anion types; Various proton hopping pathways in selected compounds; Impact of functional choice on proton hopping barrier; Distribution of metal–oxygen bond length for other element groups tested; Impact of coordination environment on proton hopping barrier; Atomic coordinates for β-Ba_2_ScAlO_5_ and β-Ba_7_Nb_4_MoO_20_ (PDF)

## #

Present Address: Department of Chemical and Materials Engineering, San Jose State University, San Jose, California 95192, United States

## References

[1] Chung H. W., Cladek B., Hsiau Y.-Y., Hu Y.-Y., Page K., Perry N. H., Yildiz B., Haile S. M. (2024). Hydrogen in energy
and information sciences. MRS Bull.

[2] Yuan Y., Patel R. K., Banik S., Reta T. B., Bisht R. S., Fong D. D., Sankaranarayanan S. K., Ramanathan S. (2024). Proton conducting
neuromorphic materials and devices. Chem. Rev..

[3] Onen M., Emond N., Wang B., Zhang D., Ross F. M., Li J., Yildiz B., Del Alamo J. A. (2022). Nanosecond protonic programmable
resistors for analog deep learning. Science.

[4] Kreuer K.-D. (2003). Proton-conducting
oxides. Annu. Rev. Mater. Res..

[5] Duan C., Huang J., Sullivan N., O’Hayre R. (2020). Proton-conducting
oxides for energy conversion and storage. Appl.
Phys. Rev..

[6] Vignesh D., Rout E. (2023). Technological challenges and advancement in proton conductors: a
review. Energy Fuels.

[7] Fop S. (2021). Solid oxide
proton conductors beyond perovskites. J. Mater.
Chem. A.

[8] Fabbri E., Pergolesi D., Traversa E. (2010). Materials challenges toward proton-conducting
oxide fuel cells: a critical review. Chem. Soc.
Rev..

[9] Nakamura T., Mizunuma S., Kimura Y., Mikami Y., Yamauchi K., Kuroha T., Taniguchi N., Tsuji Y., Okuyama Y., Amezawa K. (2018). Energy efficiency of
ionic transport through proton
conducting ceramic electrolytes for energy conversion applications. J. Mater. Chem. A.

[10] Han D., Toyoura K., Uda T. (2021). Protonated BaZr_0.8_ Y_0.2_O_3–*δ*
_: Impact of
Hydration on Electrochemical Conductivity and Local Crystal Structure. ACS Appl. Ener. Mater..

[11] Han D., Liu X., Bjørheim T. S., Uda T. (2021). Yttrium-Doped Barium
Zirconate-Cerate Solid Solution as Proton Conducting Electrolyte:
Why Higher Cerium Concentration Leads to Better Performance for Fuel
Cells and Electrolysis Cells. Adv. Ener. Mater..

[12] Vera C. Y. R., Ding H., Peterson D., Gibbons W. T., Zhou M., Ding D. (2021). A mini-review on proton conduction of BaZrO_3_-based perovskite
electrolytes. J. Phys. Energy.

[13] Han D., Nose Y., Shinoda K., Uda T. (2012). Site selectivity of
dopants in BaZr_1–*y*
_M*
_y_
*O_3–*δ*
_ (M
= Sc, Y, Sm, Eu, Dy) and measurement of their water contents and conductivities. Solid State Ionics.

[14] Rowberg A. J. E., Li M., Ogitsu T., Varley J. B. (2023). Polarons
and electrical
leakage in BaZrO_3_ and BaCeO_3_. Phys. Rev. Mater..

[15] Zhang S., Rowberg A. J., Ogitsu T., Pham T. A., Varley J. B. (2025). Electron-Phonon
Renormalization in the Proton-Conducting Electrolyte BaZrO_3_ and Its Implications for High-Temperature Electrolysis. PRX Energy.

[16] Fujii T., Toyoura K., Uda T., Kasamatsu S. (2021). Theoretical
study on proton diffusivity in Y-doped BaZrO_3_ with realistic
dopant configurations. Phys. Chem. Chem. Phys..

[17] Toyoura K., Meng W., Han D., Uda T. (2018). Preferential proton
conduction along a three-dimensional dopant network in yttrium-doped
barium zirconate: a first-principles study. J. Mater. Chem. A.

[18] Rowberg A. J. E., Weston L., Van de Walle C. G. (2019). Optimizing proton conductivity in
zirconates through defect engineering. ACS Appl.
Ener. Mater..

[19] Morin-Martinez, A. A. ; Dillenz, M. ; Sjølin, B. H. ; Dhanalakshmi, R. B. ; Vegge, T. ; Esposito, V. ; Castelli, I. E. Understanding Oxygen-Ion Migration in Hexagonal Perovskites Through Autonomous Workflows and Density Functional Theory. ChemRxiv 2025.

[20] Meng J., Sheikh M. S., Jacobs R., Liu J., Nachlas W. O., Li X., Morgan D. (2024). Computational discovery of fast interstitial oxygen
conductors. Nat. Mater..

[21] Fop S., McCombie K. S., Wildman E. J., Skakle J. M., Irvine J. T., Connor P. A., Savaniu C., Ritter C., Mclaughlin A. C. (2020). High oxide
ion and proton conductivity in a disordered hexagonal perovskite. Nat. Mater..

[22] Murakami T., Hester J. R., Yashima M. (2020). High proton conductivity
in Ba5Er2Al2ZrO13,
a hexagonal perovskite-related oxide with intrinsically oxygen-deficient
layers. J. Am. Chem. Soc..

[23] Murakami T., Avdeev M., Morikawa R., Hester J. R., Yashima M. (2023). High Proton
Conductivity in *β*-Ba2ScAlO5 Enabled by Octahedral
and Intrinsically Oxygen-Deficient Layers. Adv.
Funct. Mater..

[24] Lu N., Zhang Z., Wang Y., Li H.-B., Qiao S., Zhao B., He Q., Lu S., Li C., Wu Y. (2022). Enhanced low-temperature proton conductivity in hydrogen-intercalated
brownmillerite oxide. Nat. Energy.

[25] Fop S., Dawson J. A., Fortes A. D., Ritter C., McLaughlin A. C. (2021). Hydration
and ionic conduction mechanisms of hexagonal perovskite derivatives. Chem. Mater..

[26] Waroquiers D., Gonze X., Rignanese G.-M., Welker-Nieuwoudt C., Rosowski F., Gobel M., Schenk S., Degelmann P., André R., Glaum R. (2017). Statistical
analysis
of coordination environments in oxides. Chem.
Mater..

[27] Jain A., Ong S. P., Hautier G., Chen W., Richards W. D., Dacek S., Cholia S., Gunter D., Skinner D., Ceder G. (2013). Commentary: The Materials Project: A materials genome
approach to accelerating materials innovation. APL Mater..

[28] Hohenberg P., Kohn W. (1964). Inhomogeneous electron gas. Phys. Rev..

[29] Kohn W., Sham L. J. (1965). Self-Consistent
Equations Including Exchange and Correlation
Effects. Phys. Rev..

[30] Kresse G., Hafner J. (1993). Ab initio molecular
dynamics for liquid metals. Phys. Rev. B.

[31] Kresse G., Hafner J. (1994). Ab initio molecular-dynamics
simulation of the liquid-metal–amorphous-semiconductor
transition in germanium. Phys. Rev. B.

[32] Kresse G., Furthmüller J. (1996). Efficiency
of ab-initio total energy calculations for
metals and semiconductors using a plane-wave basis set. Comput. Mater. Sci..

[33] Kresse G., Furthmüller J. (1996). Efficient iterative schemes for ab
initio total-energy
calculations using a plane-wave basis set. Phys.
Rev. B.

[34] Perdew J. P., Burke K., Ernzerhof M. (1996). Generalized
gradient approximation
made simple. Phys. Rev. Lett..

[35] Blöchl P. E. (1994). Projector
augmented-wave method. Phys. Rev. B.

[36] Kresse G., Joubert D. (1999). From ultrasoft pseudopotentials
to the projector augmented-wave
method. Phys. Rev. B.

[37] Van
de Walle C. G., Neugebauer J. (2006). Hydrogen in semiconductors. Annu. Rev. Mater. Res..

[38] Zhang L., Yao F., Meng J., Zhang W., Wang H., Liu X., Meng J., Zhang H. (2019). Oxygen migration and proton diffusivity
in transition-metal (Mn, Fe, Co, and Cu) doped Ruddlesden–Popper
oxides. J. Mater. Chem. A.

[39] Henkelman G., Uberuaga B. P., Jónsson H. (2000). A climbing
image nudged elastic band
method for finding saddle points and minimum energy paths. J. Chem. Phys..

[40] Henkelman G., Jónsson H. (2000). Improved tangent estimate in the nudged elastic band
method for finding minimum energy paths and saddle points. J. Chem. Phys..

[41] Žguns P., Klyukin K., Wang L. S., Xiong G., Li J., Haile S. M., Yildiz B. (2024). Uncovering fast solid-acid proton
conductors based on dynamics of polyanion groups and proton bonding
strength. Energy Environ. Sci..

[42] Fop S., Dawson J. A., Tawse D. N., Skellern M. G., Skakle J. M., Mclaughlin A. C. (2022). Proton
and oxide ion conductivity in palmierite oxides. Chem. Mater..

[43] Mó O., Yáñez M., Eckert-Maksić M., Maksić Z. B., Alkorta I., Elguero J. (2005). Periodic trends in bond dissociation
energies. A theoretical study. J. Phys. Chem.
A.

[44] Pearson R.
G. (1986). Absolute
electronegativity and hardness correlated with molecular orbital theory. Proc. Natl. Acad. Sci. U. S. A..

[45] García-González E., Parras M., González-Calbet J. (1999). Crystal structure of
an unusual polytype: 7H-Ba7Nb4MoO20. Chem. Mater..

[46] Aroyo M. I., Perez-Mato J. M., Capillas C., Kroumova E., Ivantchev S., Madariaga G., Kirov A., Wondratschek H. (2006). Bilbao Crystallographic
Server: I. Databases and crystallographic computing programs. Z. Kristallogr Cryst. Mater..

[47] Aroyo M. I., Kirov A., Capillas C., Perez-Mato J., Wondratschek H. (2006). Bilbao Crystallographic Server. II.
Representations
of crystallographic point groups and space groups. Acta Crystallogr. A.

[48] Momma K., Izumi F. (2011). VESTA3 for three-dimensional visualization
of crystal, volumetric
and morphology data. J. Appl. Crystallogr..

[49] Youn Y., Hussain B., Ullah A., Hwang I. J., Shin J., Hong J.-E., Joh D. W., Lee S.-B., Song R.-H., Park S.-J. (2023). Anisotropic
proton migration in hexagonal perovskite-related
Ba5Er2Al2ZrO13 oxide. Chem. Mater..

[50] Zhou Y., Fop S., Mclaughlin A. C., Dawson J. A. (2025). Elucidating oxide-ion
and proton transport in ionic conductors using machine learning potentials. Npj Comput. Mater..

[51] Sun J., Ruzsinszky A., Perdew J. P. (2015). Strongly constrained and appropriately
normed semilocal density functional. Phys. Rev.
Lett..

[52] Yashima M., Tsujiguchi T., Sakuda Y., Yasui Y., Zhou Y., Fujii K., Torii S., Kamiyama T., Skinner S. J. (2021). High oxide-ion
conductivity through the interstitial oxygen site in Ba7Nb4MoO20-based
hexagonal perovskite related oxides. Nat. Commun..

