# Supplementary material for: First-Principles Evaluation of Proton Hopping in Tetrahedral Oxide Motifs
Source: Chem Mater. 2026 Feb 23;38(5):2227–36. doi: 10.1021/acs.chemmater.5c02422 (PMC12980716; doi:10.1021/acs.chemmater.5c02422)
Supplement: Supplementary file 1 [file cm5c02422_si_001.pdf]

Supporting Information for "First-principles evaluation of proton hopping in tetrahedral oxide motifs"

Shenli Zhang,[*,†,¶] Andrew J. E. Rowberg,[†] ShinYoung Kang,[†] and Joel B. Varley[*,†]

†*Quantum Simulations Group, Materials Science Division, Lawrence Livermore National Laboratory, Livermore, California 94550, U.S.A*

‡*Laboratory for Energy Applications for the Future, Lawrence Livermore National Laboratory, Livermore, California 94550, U.S.A*

¶*Present Address: Department of Chemical and Materials Engineering, San Jose State University, San Jose, California, 95192, United States*

E-mail: shenli.zhang@sjsu.edu; varley2@llnl.gov

Table S1: **Equilibrium M–O bond length for each metal element**: computed by PBE functional using zinc blende structure.

Element	Al	Ga	In	Si	Ge	Sn	V	Cr	Co	Co	Ni	Nb	Mo	W	Sc	Y	Zr
Bond length (Å)	1.64	1.80	1.97	1.41	1.60	1.72	1.53	1.39	1.63	1.45	1.65	1.73	1.42	1.61	1.92	2.10	1.77
Valence	3+	3+	3+	4+	4+	4+	5+	5+	3+	4+	3+	5+	6+	6+	3+	3+	4+

Table S2: **Linear fitting parameters**: proton hopping barrier change (y) as a function of M-O bond length (x), described by y=ax+b. The x-axis intercept -b/a and square of the correlation coefficient R^2 value are also given. We note for some elements, the data points at longer bond length may deviate from the linear behavior due to the decrease of bonding strength, and such deviation decreases the R^2 value.

Element	Al	Ga	In	Si	Ge	Sn	V	Cr	Co	Co	Ni	Nb	Mo	W	Sc	Y	Zr
a (eV/Å)	1.82	2.00	3.55	1.58	2.15	2.40	1.44	1.48	6.28	2.21	3.74	2.40	1.32	1.57	1.56	2.54	2.19
b (eV)	-2.85	-3.22	-6.73	-2.35	-3.37	-4.01	-2.38	-2.52	-10.84	-3.58	-6.49	-4.13	-2.24	-2.67	-2.59	-4.82	-4.82
-b/a (Å)	1.57	1.61	1.90	1.48	1.56	1.67	1.65	1.70	1.73	1.62	1.74	1.72	1.69	1.71	1.66	1.90	1.74
R^2	0.98	0.89	0.99	0.89	0.96	0.94	0.99	1.00	0.96	0.97	0.97	1.00	0.96	0.95	0.81	0.92	0.88

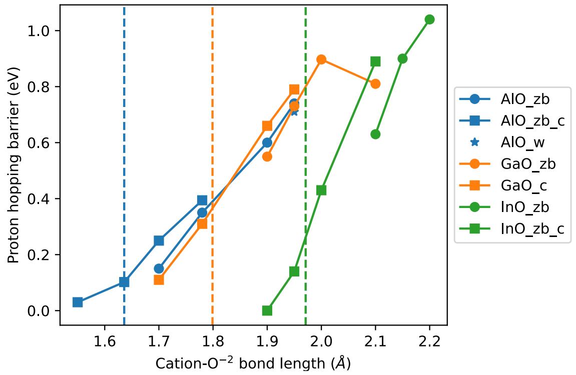

Figure S1: Comparing proton hopping barrier computed by fixing majority of cation ions (labeled as "c" in the figure) versus complete relaxation in the cell. For AlO case, the comparison between the values obtained in the zinc blende structure (labeled as "zb" in the figure) and in the wurtzite structure (labeled as "w" in the figure) is also shown. Vertical dashed lines are the equilibrium M-O bond lengths obtained in the crystal structure without protons.

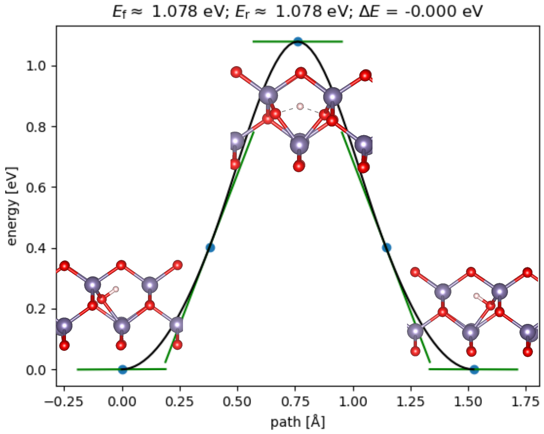

Figure S2: Potential energy change along the proton hopping pathway in the zinc blende crystal structure calculated with NEB method. Shown for GeO case as an example. Color scheme: purple: Ge; red: O; white: H.

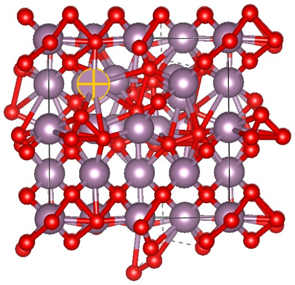

Figure S3: A MoO configuration in which tetrahedral units are distorted, because of the too short Mo-O bond length.

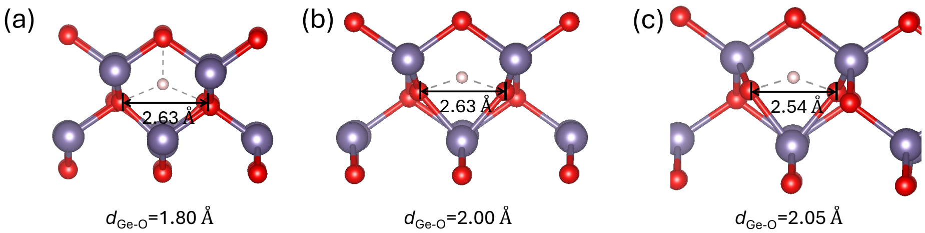

Figure S4: Increased M–O bond bending and non-monotonic change of O–O distance during proton hopping at different M–O bond length, using GeO case as an example. Ge–O bond length equals to (a) 1.80 Å (b) 2.00 Å. (c) 2.05 Å.

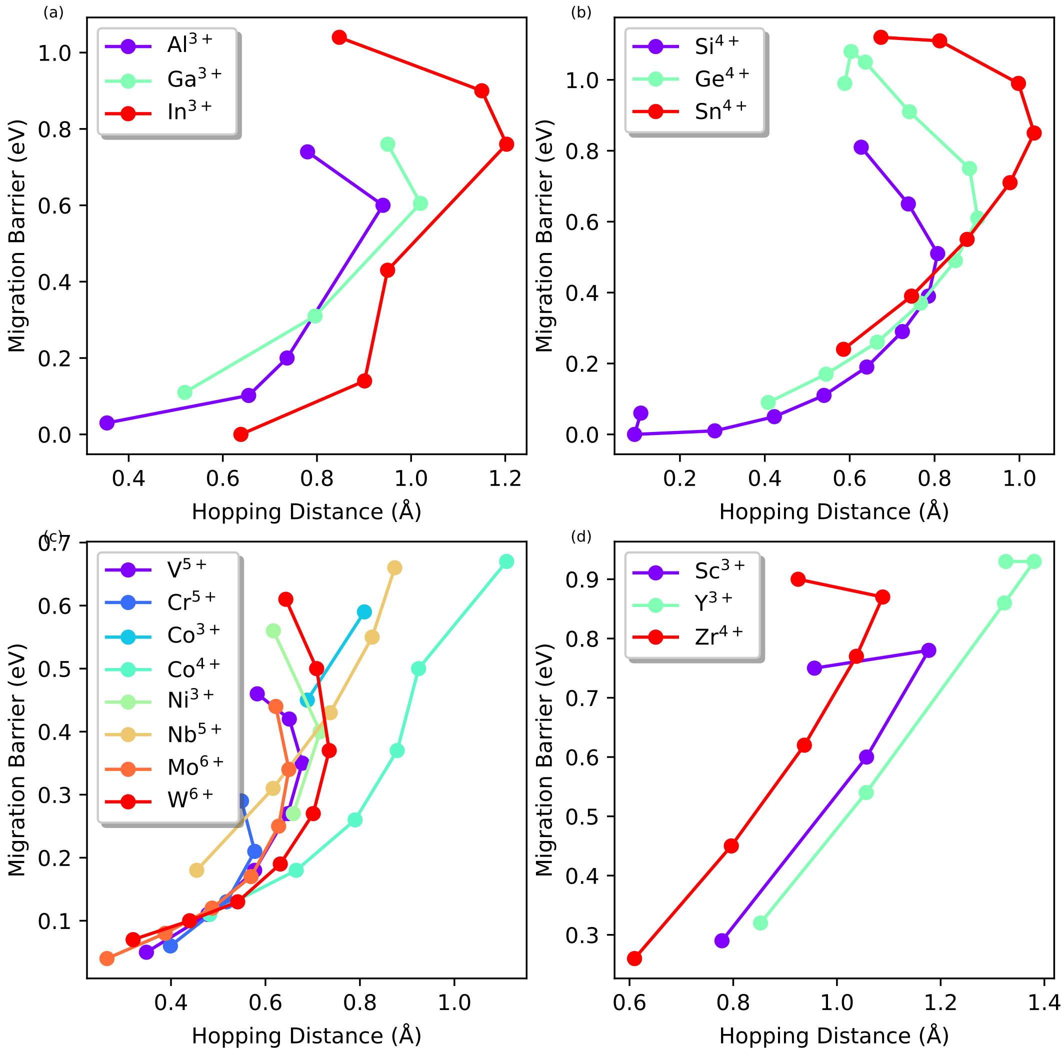

Figure S5: Proton hopping barrier as a function of hopping distance, which is calculated as the distance between the initial and final proton coordinates.

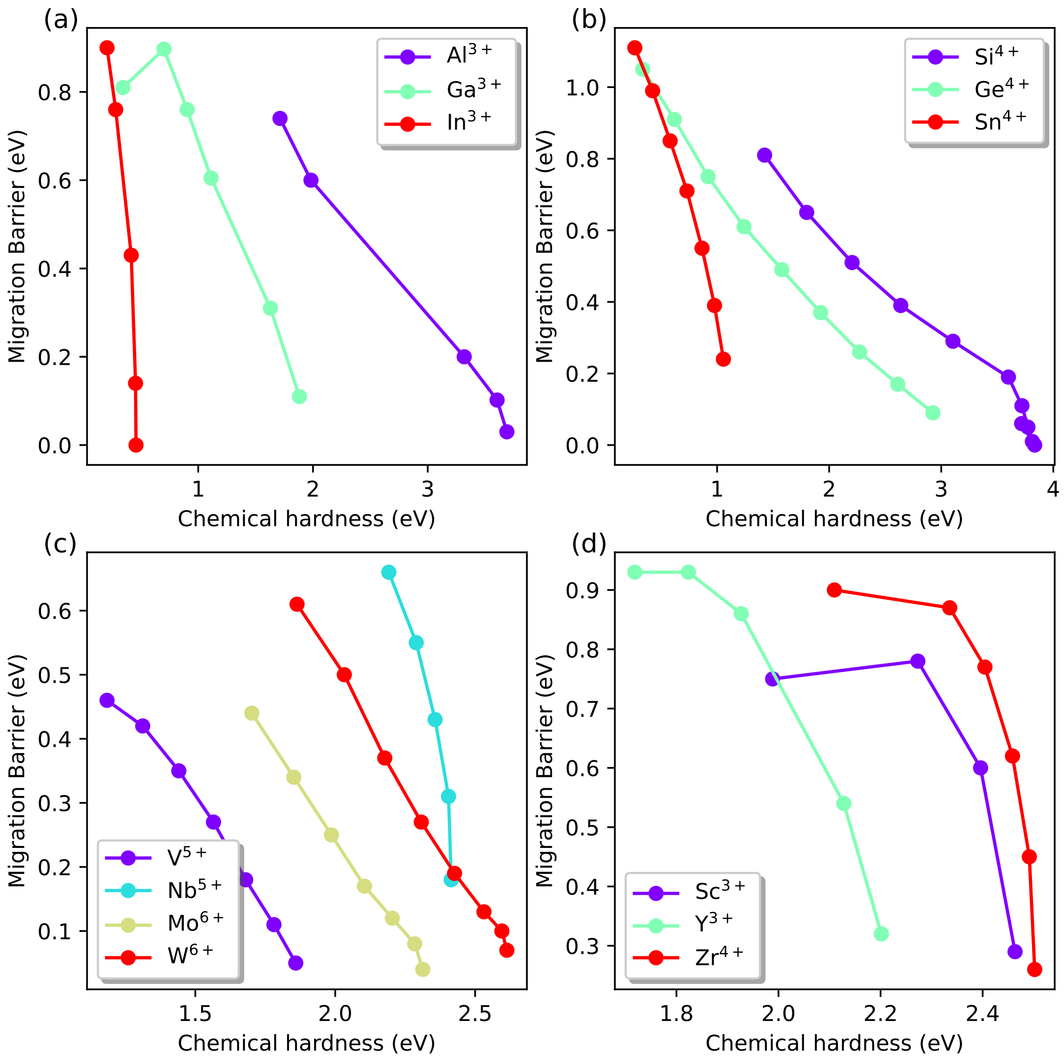

Figure S6: Proton hopping barrier as a function of chemical hardness, which is calculated as twice the band gap energy. We note despite the band gap energy may not be realistic in our motif structures (idealized, simplified crystal structures with potential underestimation with PBE functional), the qualitative trend indeed reveals the correlation between proton hopping barrier and the chemical hardness. We also note not every case is insulating, and we exclude those on this plot (Cr^{5+}, Co^{3+}, Co^{4+} and Ni^{3+}).

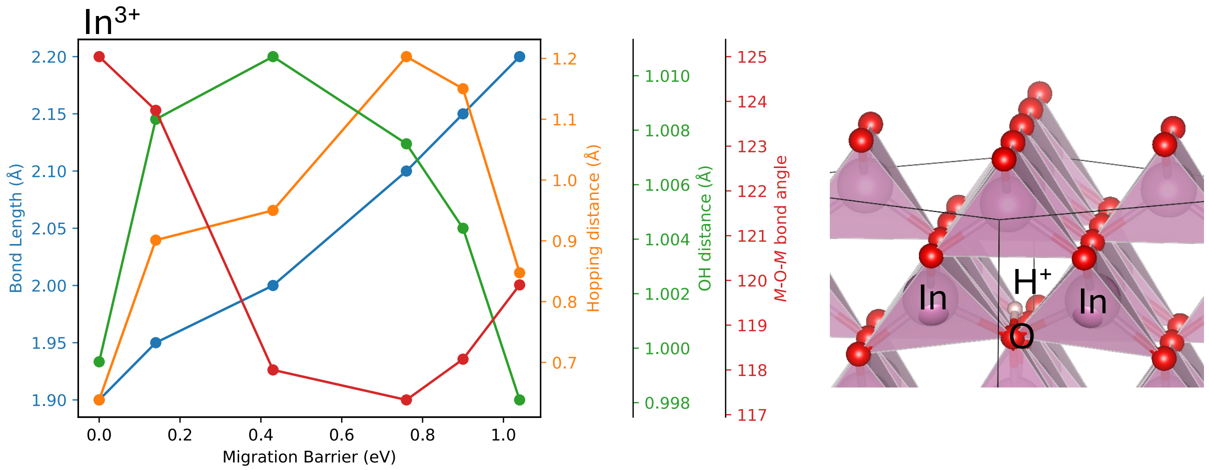

Figure S7: Various descriptors change (M-O bond length, hopping distance, O-H distance and M-O-M bond angle) as a function of the proton migration barrier for In^{3+} as an example. The corresponding In-O-In angle is marked on the right panel.

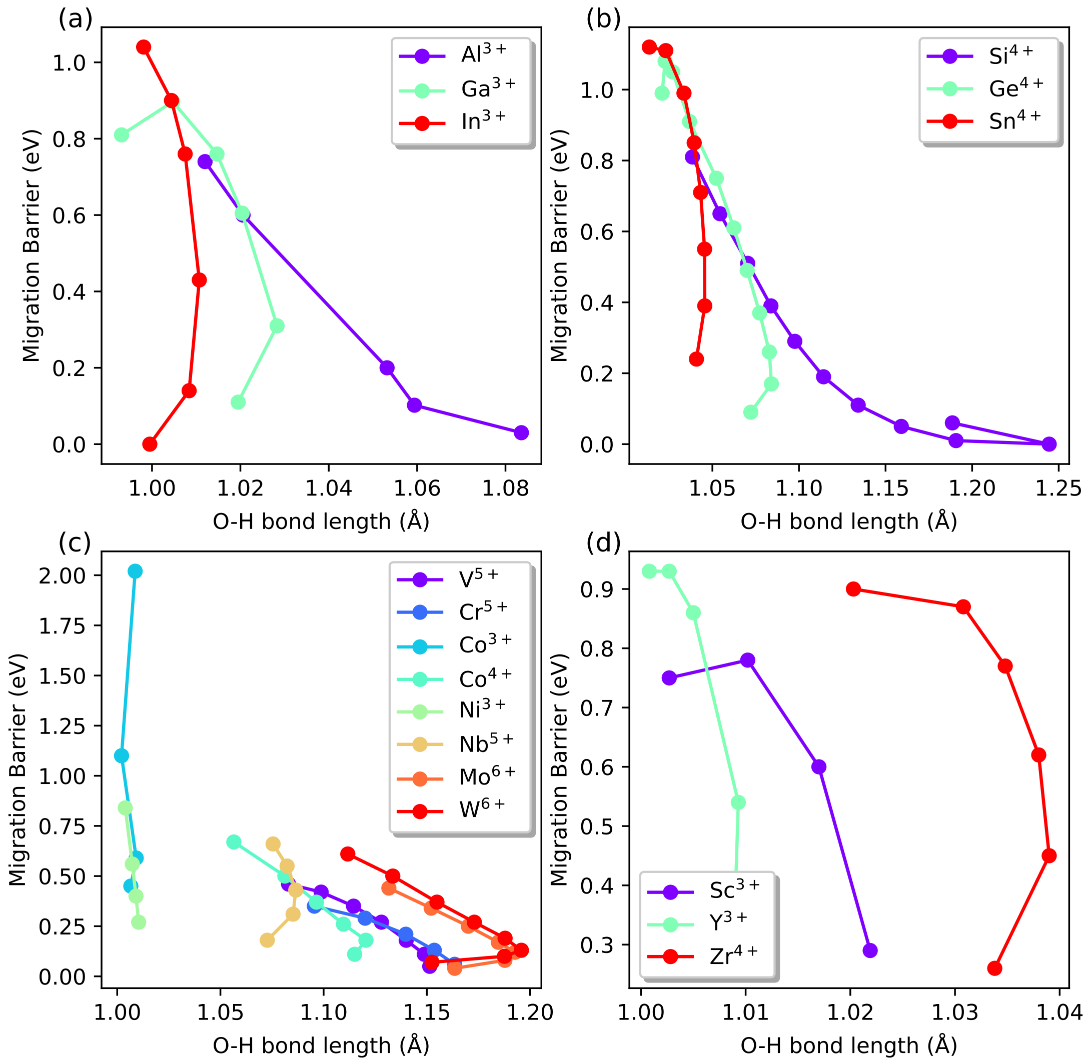

Figure S8: Proton hopping barrier as a function of O–H bond length.

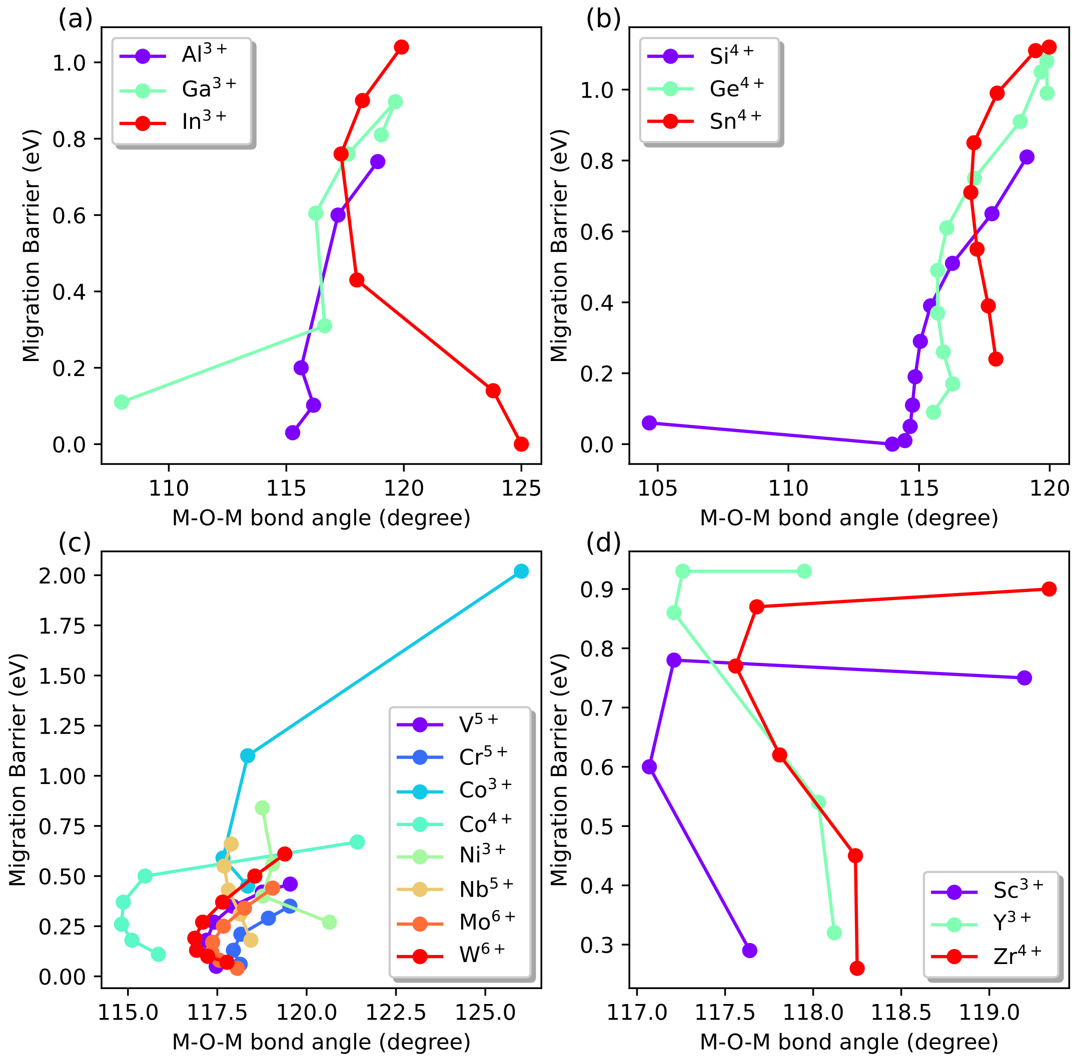

Figure S9: Proton hopping barrier as a function of M-O-M bond angle.

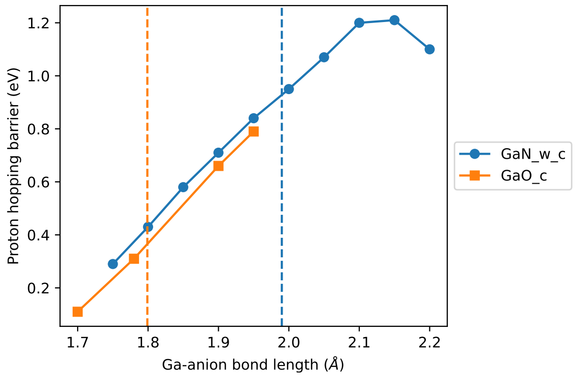

Figure S10: Comparing proton hopping barrier around Ga^{3+} tetrahedral units with different anion types: O^{2-} and N^{3-}.Vertical dashed lines are the equilibrium M-O bond lengths obtained in the crystal structure without protons.

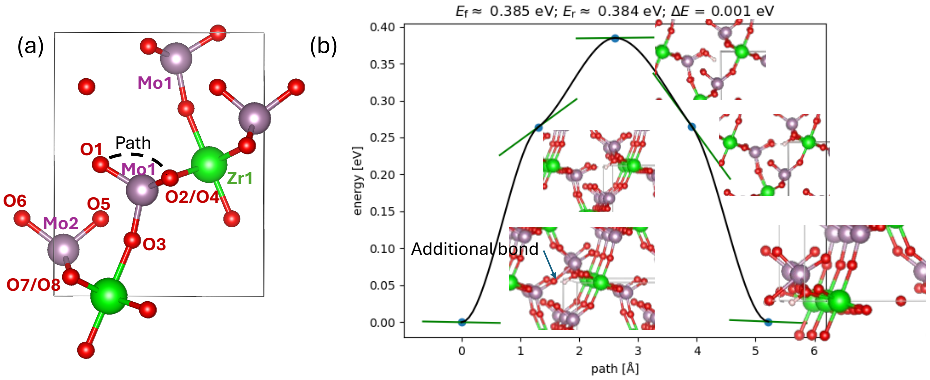

Figure S11: (a) Symmetrically nonequivalent Mo sites and the associated possible proton hopping pathways in $Zr(MoO_4)_2$. (b) One proton hopping pathway around Mo_2 site with a fifth Mo–O bond formation.

Two different Mo sites exist in the unit cell (denoted as Mo_1 and Mo_2) and as the Mo–O bond length varies between 1.70 and 1.78 Å, we identified seven different intra-hopping pathways(as detailed in Fig. S11 and Table S4). Similar to the zinc blende structure case, we fixed all the cation sites except the ones bonded to oxygen sites that are involved during proton hopping. The same is applied for $Ba_3V_2O_8$ case, which may lead to overestimation of higher proton hopping barriers as discussed in the Methods section in the main manuscript.

Table S3: **Proton hopping pathways and associated hopping barrier in $Zr(MoO_4)_2$.**

Path	d_{O-O} (Å)	d_{Mo-O} (Å)	Barrier (eV)
O1–O2	2.87	1.70, 1.78	-
O2–O3	2.91	1.77, 1.78	0.5
O2–O4	2.87	1.78	-
O5–O6	2.74	1.69, 1.81	-
O5–O7	2.73	1.69, 1.80	-
O7–O8	3.00	1.80	0.38
O6–O7	3.06	1.80, 1.81	0.45

*For pathways without the barrier values, no appropriate convergence of NEB paths could be identified.

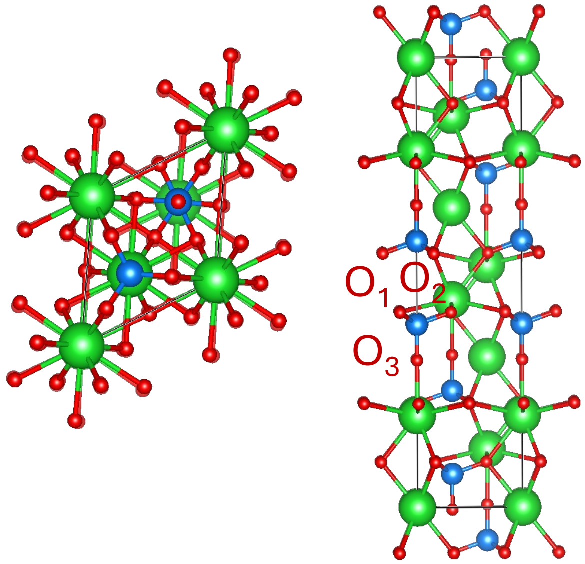

Figure S12: Symmetrically nonequivalent oxygen sites for proton hopping in $Ba_3V_2O_8$. Only one type of V site is available in this $R\overline{3}m$ crystal symmetry. Color scheme: green: Ba, blue: V, red: O.

Table S4: **Proton hopping pathways and associated hopping barrier in $Ba_3V_2O_8$.**

Path	d_{O-O} (Å)	d_{V-O} (Å)	Barrier (eV)
O1–O2	2.812	1.72	1.39
O1–O3	2.806	1.72, 1.71	1.1

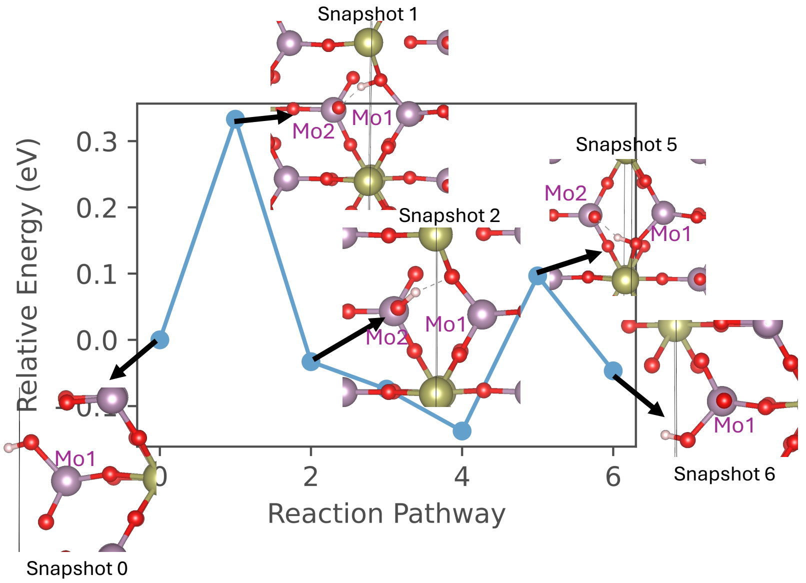

Figure S13: Proton (white color) hopping between two oxygen sites in one tetrahedral MoO_4 unit (Mo1) involves an intermediate hopping to oxygen site in the neighboring MoO_4 unit (Mo2) in $Hf(MoO_4)_2$ compound.

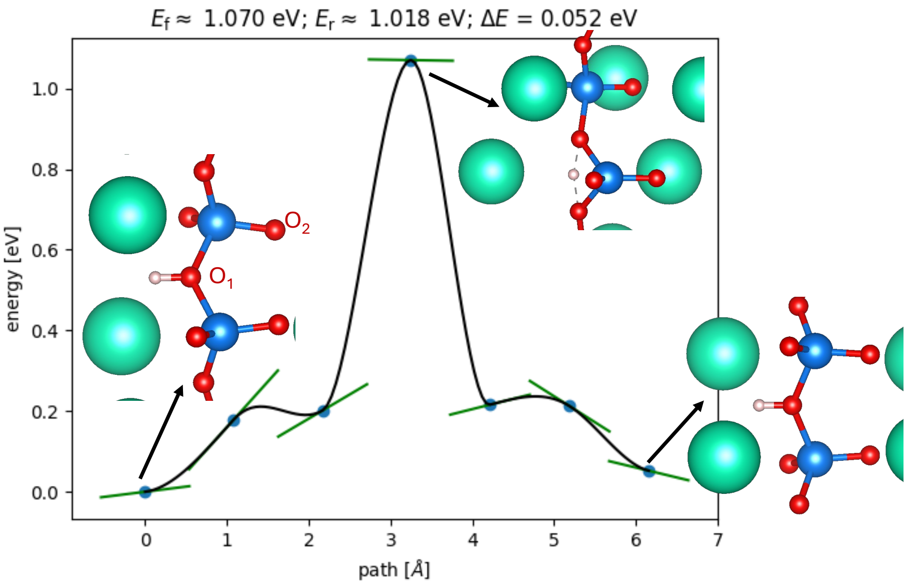

Figure S14: Proton hopping pathway corresponding to the 1.04 eV barrier in $CsVO_3$. In the material, there are two symmetry inequivalent oxygen sites: O_1 is shared between two V sites, while O_2 is shared between Cs sites. Hopping pathway involving O_2 leads to higher barrier about 2 eV.

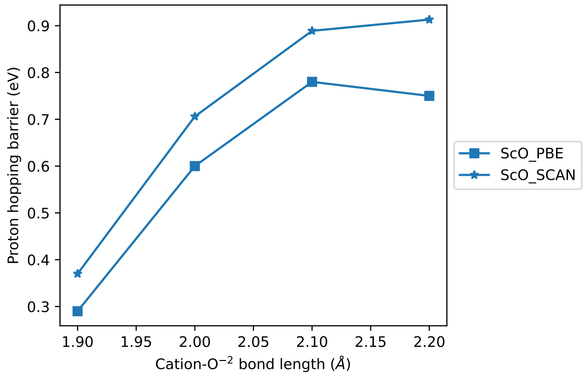

Figure S15: Comparing proton hopping barrier computed with PBE functional and with SCAN functional for ScO zinc blende structure.

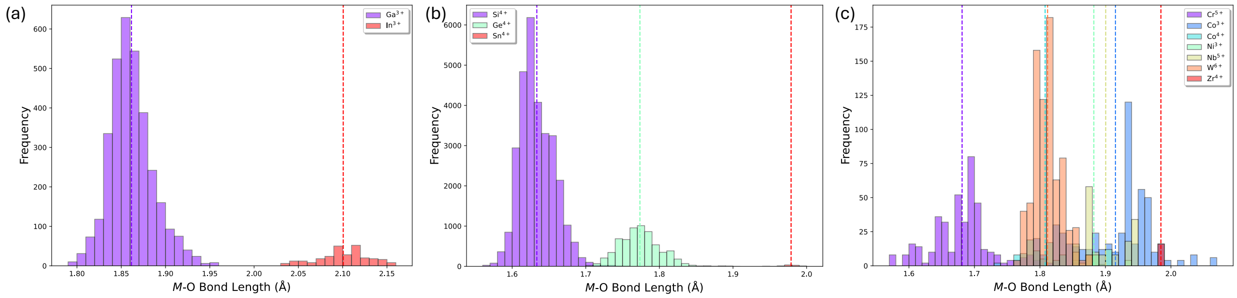

Figure S16: Distribution of M-O bond lengths in tetrahedral units in real materials based on crystal structure database.[1,2] (a) Boron group elements. (b) Group IV elements. (c) Transition metals group.

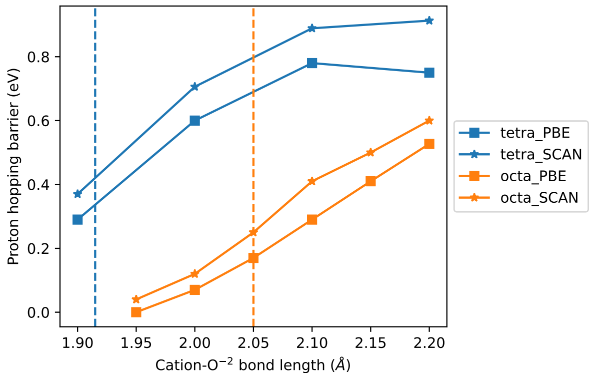

Figure S17: Comparison of proton hopping barriers in tetrahedral and octahedral units for Sc^{3+} case. The octahedral unit is implemented in a rocksalt structure.

β-Ba_2ScAlO_5: generated by bilbao crystallographic server

1.00000000000000

5.8021898270	0.0000000000	0.0000000000
-2.9010949135	5.0248437877	0.0000000000
0.0000000000	0.0000000000	19.5911006927

Ba Al Sc O

8 4 4 20

Direct

0.000000000	0.000000000	0.000000000
0.000000000	0.000000000	0.500000000
0.333333343	0.666666687	0.750000000
0.666666627	0.333333313	0.250000000
0.333333343	0.666666687	0.385329992
0.666666627	0.333333313	0.614670038
0.666666627	0.333333313	0.885329962
0.333333343	0.666666687	0.114670008
0.000000000	0.000000000	0.164940000
0.000000000	0.000000000	0.835060000
0.000000000	0.000000000	0.664940000
0.000000000	0.000000000	0.335060000
0.333333343	0.666666687	0.565890014
0.666666627	0.333333313	0.434109986
0.666666627	0.333333313	0.065890014
0.333333343	0.666666687	0.934109986
0.500000000	0.000000000	0.000000000
0.000000000	0.500000000	0.000000000
0.500000000	0.500000000	0.000000000
0.500000000	0.000000000	0.500000000
0.000000000	0.500000000	0.500000000

0.500000000	0.500000000	0.500000000
0.162939996	0.325870007	0.629540026
0.837059975	0.674129963	0.370459974
0.674129963	0.837069988	0.629540026
0.325870007	0.162930012	0.370459974
0.162930012	0.837059975	0.629540026
0.837069988	0.162939996	0.370459974
0.837059975	0.674129963	0.129540026
0.162939996	0.325870007	0.870459974
0.325870007	0.162930012	0.129540026
0.674129963	0.837069988	0.870459974
0.837069988	0.162939996	0.129540026
0.162930012	0.837059975	0.870459974
0.000000000	0.000000000	0.250000000
0.000000000	0.000000000	0.750000000

β-$Ba_7Nb_4MoO_{20}$: generated by bilbao crystallographic server

1.0		
5.86440000	0.0000000000	0.0000000000
0.0000000000	5.86440000	0.0000000000
0.0000000000	0.0000000000	16.527240000
Ba	Nb Mo	O
7	4 1	20
Direct		

0.000000000	0.000000000	0.000000000
0.333333333	0.666666667	0.823660000
0.666666667	0.333333333	0.176340000
0.000000000	0.000000000	0.281260000
0.000000000	0.000000000	0.718740000
0.333333333	0.666666667	0.576970000
0.666666667	0.333333333	0.423030000
0.333333333	0.666666667	0.094350000
0.666666667	0.333333333	0.905650000
0.333333333	0.666666667	0.349350000
0.666666667	0.333333333	0.650650000
0.000000000	0.000000000	0.500000000
0.173870000	0.826130000	0.131630000
0.173870000	0.347740000	0.131630000
0.652260000	0.826130000	0.131630000
0.347740000	0.173870000	0.868370000
0.826130000	0.173870000	0.868370000
0.826130000	0.652260000	0.868370000
0.333333333	0.666666667	0.989430000
0.666666667	0.333333333	0.010570000
0.163880000	0.836120000	0.432330000
0.163880000	0.327760000	0.432330000

0.672240000	0.836120000	0.432330000
0.327760000	0.163880000	0.567670000
0.836120000	0.163880000	0.567670000
0.836120000	0.672240000	0.567670000
0.500000000	0.000000000	0.293930000
0.000000000	0.500000000	0.293930000
0.500000000	0.500000000	0.293930000
0.500000000	0.000000000	0.706070000
0.000000000	0.500000000	0.706070000
0.500000000	0.500000000	0.706070000

References

(1) Waroquiers, D.; Gonze, X.; Rignanese, G.-M.; Welker-Nieuwoudt, C.; Rosowski, F.; Gobel, M.; Schenk, S.; Degelmann, P.; André, R.; Glaum, R., et al. Statistical analysis of coordination environments in oxides. *Chem. Mater.* **2017**, *29*, 8346–8360.

(2) Jain, A.; Ong, S. P.; Hautier, G.; Chen, W.; Richards, W. D.; Dacek, S.; Cholia, S.; Gunter, D.; Skinner, D.; Ceder, G.; Persson, K. A. Commentary: The Materials Project: A materials genome approach to accelerating materials innovation. *APL Materials* **2013**, *1*, 011002.
